# The Impact of Metabolic Rewiring in Glioblastoma: The Immune Landscape and Therapeutic Strategies

**DOI:** 10.3390/ijms26020669

**Published:** 2025-01-14

**Authors:** Yuganthini Vijayanathan, Ivy A. W. Ho

**Affiliations:** 1 Molecular Neurotherapeutics Laboratory, National Neuroscience Institute, Singapore 308433, Singapore; yuganthini_p_vijayanathan@nni.com.sg; 2 Duke-NUS Medical School, Singapore 169857, Singapore; 3 Department of Physiology, National University of Singapore, Singapore 117593, Singapore

**Keywords:** metabolism, glioma, GBM, tumor microenvironment, immune infiltration

## Abstract

Glioblastoma (GBM) is an aggressive brain tumor characterized by extensive metabolic reprogramming that drives tumor growth and therapeutic resistance. Key metabolic pathways, including glycolysis, lactate production, and lipid metabolism, are upregulated to sustain tumor survival in the hypoxic and nutrient-deprived tumor microenvironment (TME), while glutamine and tryptophan metabolism further contribute to the aggressive phenotype of GBM. These metabolic alterations impair immune cell function, leading to exhaustion and stress in CD8+ and CD4+ T cells while favoring immunosuppressive populations such as regulatory T cells (Tregs) and M2-like macrophages. Recent studies emphasize the role of slow-cycling GBM cells (SCCs), lipid-laden macrophages, and tumor-associated astrocytes (TAAs) in reshaping GBM’s metabolic landscape and reinforcing immune evasion. Genetic mutations, including *Isocitrate Dehydrogenase* (*IDH*) mutations, *Epidermal Growth Factor Receptor* (*EGFR*) amplification, and *Phosphotase and Tensin Homolog* (*PTEN*) loss, further drive metabolic reprogramming and offer potential targets for therapy. Understanding the relationship between GBM metabolism and immune suppression is critical for overcoming therapeutic resistance. This review focuses on the role of metabolic rewiring in GBM, its impact on the immune microenvironment, and the potential of combining metabolic targeting with immunotherapy to improve clinical outcomes for GBM patients.

## 1. Introduction

GBM is one of the most challenging tumors to treat, with a median survival of less than 2 years [1]. Its TME is highly heterogeneous, characterized by metabolic diversity, genetic modulation, immune cell interactions, and various microenvironmental factors [2,3,4,5]. The Ivy Glioblastoma Atlas Project (IVYGAP) has identified five anatomically distinct structures of GBM: leading edge, tumor-infiltrating, cellular tumor, microvascular proliferation, and pseudopalisading cells around necrosis. These regions can be characterized into specialized niches: perivascular (microvascular proliferation), hypoxia–necrotic (cellular tumor, pseudopalisading cells around necrosis), and invasive (leading edge and tumor-infiltrating) [6]. This molecular heterogeneity is apparent in a recent study that reported differential expression of programmed death-ligand 1 (PD-L1) protein within the different tumor areas (core, periphery, and healthy tissue) in the same GBM patient [7].

As GBM progresses, insufficient oxygen leads to hypoxic regions that induce pseudopalisading necrosis. Hypoxia triggers various adaptive mechanisms in GBM, including the upregulation of genes involved in metabolism and immune evasion, as well as alterations in angiogenesis. The abnormal vascularization resulting from the activation of Hypoxia-Inducible Factor 1 subunit α (HIF1α)-responsive genes, such as vascular endothelial growth factor (VEGF) and interleukin (IL)-8, combined with a disrupted blood–brain barrier (BBB), facilitates immune cell infiltration, which exacerbates GBM growth. At the tumor leading edge, GBM cells exhibit enhanced invasion due to a functional vasculature that supplies nutrients and oxygen. Additionally, these tumor cells interact with surrounding immune cells and the extracellular matrix (ECM) to establish a pro-tumoral microenvironment [8]. Glioma stem cells (GSCs) located at the leading edge often exhibit metabolic plasticity distinct from those in the hypoxic core, contributing to their invasiveness and therapeutic resistance [9]. Overall, the GBM TME is highly dynamic, with the leading and infiltrating edges being critical for tumor progression, making therapeutic strategies targeting these regions essential for improving patient outcomes.

Despite extensive research on the metabolic alterations driving GBM progression or the immune suppression within its TME, the intricate interplay between these domains remains underexplored. This review investigates the complexity of the TME, emphasizing the dynamic interactions between hypoxia, immune modulation, and metabolic plasticity and their synergistic role in shaping GBM’s aggressive and therapy-resistant phenotype. It also highlights emerging contributors such as SCCs, lipid-laden macrophages, and tumor-associated astrocytes (TAAs), further enhancing the diversity and adding to the TME complexity of GBM’s metabolic and immune landscape. By highlighting the genetic alterations, including *IDH* mutations, *EGFR* amplification, and *PTEN* loss, that drive these metabolic changes, this review provides insights into the nuanced understanding of the convergence between genetic and metabolic reprogramming. Finally, it discusses the potential of targeting metabolic vulnerabilities with immunotherapies as a promising strategy to overcome therapeutic resistance, offering a comprehensive and translational resource for identifying new therapeutic approaches to GBM treatment.

## 2. GBM Metabolism

Adenosine triphosphate (ATP) is the energy medium of cell exchange. ATP hydrolysis and relevant enzymatic reactions produce the fundamental free energy to conduct biological processes essential for survival. The brain constitutes only 2% of the total body weight but consumes approximately 20% of the body’s total oxygen consumption and 60% of the daily glucose intake [10], despite having limited capability to store glucose. Neurons primarily have a high rate of oxidative metabolism, while astrocytes have higher rates of glycolysis [11,12]. Although the healthy human brain depends predominantly on glucose as the primary energy substrate, it can also derive bioenergetics from other sources—acetate, amino acid, and lipids—to fuel cellular processes.

**Figure 1:**
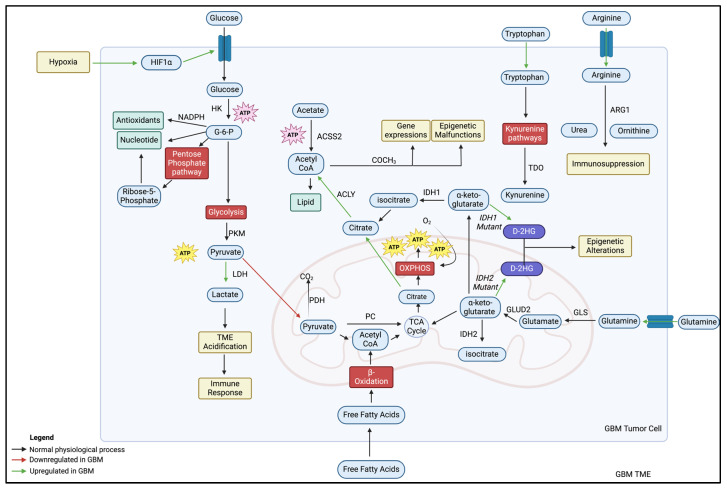
The altered GBM metabolism. The upregulation of HIF1α promotes glucose uptake via GLUT1 at the plasma membrane due to hypoxia. The initial step of glucose via the glycolysis pathway is catalyzed by HK to produce G-6-P and diverted into other pathways to synthesize nucleotides and antioxidants. In the absence of oxygen and the upregulation of aerobic glycolysis in GBM cells, pyruvate is shunted away from mitochondria and metabolized to lactate and ATP via LDH. The augmentation of lactate acidifies the TME, engendering a critical immune response. A fraction of pyruvate enters the TCA cycle in the mitochondria, which shunts Acetyl CoA and citrate for lipid synthesis. In the pentose phosphate pathway, ribose-5-phosphate and NADPH are synthesized via G-6-P for nucleotide synthesis. Acetate, another primary carbon source, can produce Acetyl CoA through ACSS2 enzymatic reactions. Free fatty acids that freely cross the BBB enter GBM tumor cells, giving rise to ATP and Acetyl CoA via β-oxidation in the mitochondria. Augmented glutamine metabolism is observed in GBM tumor cells. Glutamine is metabolized into glutamate via glutaminase. Glutamine-derived α-ketoglutarate can then enter the TCA cycle in the mitochondria, which creates an electrochemical gradient for ATP production. Decreased serum levels of tryptophan in GBM patients suggest an increase in tryptophan by GBM tumor cells and kynurenine metabolism. Kynurenine, synthesized via the kynurenine pathways, is catalyzed by TDO and causes immunosuppression. Arginine metabolism involves arginase enzymatic reaction into urea and ornithine. In GBM, there is an upregulation of arginine transporters and ARG1. IDH1 and IDH2 enzymes convert α-ketoglutarate into isocitrate in the cytoplasm and mitochondria, respectively. However, mutation of these enzymes produces oncometabolite D-2HG, spawning malignant epigenetic alterations.

By contrast, compared to healthy neurons, GBM tumor cells require more energy and materials for cellular construction, signaling, managing oxidative stress, immune suppression, and other functions. The discovery of the Warburg effect has revealed that GBM can modify its metabolic phenotypes to enhance its capacity to grow, proliferate, and survive under harsh conditions. This review, therefore, explores the different metabolic modifications in the GBM TME (Figure 1).

### 2.1. ATP Production with Alternate Sources

#### 2.1.1. Glucose

Glucose is a monosaccharide with six carbons, an aldehyde group, and over 95% ATP supply in the healthy brain. As in most cancers, tumor cells rapidly and efficiently take up glucose relative to the surrounding non-tumor cells. This feature has been exploited in diagnostic imaging, such as using glucose analogs ^18^F-deoxyglucose (FDG) for positron emission tomography (PET) [13] or by administering ^13^C-glucose for magnetic resonance spectroscopy (MRS), which can differentiate aerobic and anaerobic glucose metabolism by detecting [^4−13^C]-glutamate (Glu C4) and [^3−13^C]-lactate (Lac C3) [14].

Glucose uptake by GBM cells is enhanced by the upregulation of glucose transporters (GLUT1) on the plasma membrane, potentially through hyperactivation of the phosphoinositide 3-kinase (PI3K)-protein kinase B (AKT) pathway [15], as well as through the augmentation of hexokinase (HK), HIF1α, and HIF2α [16]. Additionally, translocation of phosphorylated pyruvate kinase (PKM)-2 and c-Myc proto-oncogene induction [17] further promote glucose uptake and glycolysis. Once glucose is taken up, HK catalyzes the initial step of glycolysis by phosphorylating glucose to produce glucose-6-phosphate (G-6-P), which is then diverted into other pathways to generate antioxidants and nucleotides. Downstream in the glycolysis pathway, phosphorylation of phosphoenolpyruvate (PEP) by PKM produces pyruvate. In the absence of oxygen, pyruvate is converted to lactate by lactate dehydrogenase (LDH). Interestingly, GBM tumor cells outside the hypoxic core preferentially convert pyruvate to lactate, a process known as aerobic glycolysis or the Warburg effect. Initially, Otto Warburg reported that aerobic glycolysis results from mitochondrial dysfunction [18]. However, a recent study has demonstrated that aerobic glycolysis could still occur in tumor cells with intact mitochondria [19], raising the question, why do GBM cells, which have a high energy demand, preferentially favor the less efficient aerobic glycolysis over oxidative phosphorylation (OXPHOS), which produce 36 ATP molecules per glucose molecule? Although the significance of this phenomenon is yet to be fully elucidated, it is most likely to be a multifactorial phenomenon.

In healthy, non-proliferating cells, pyruvate is converted into citrate and enters the tricarboxylic (TCA) cycle for ATP generation. However, in normal and GBM tumor cells, Acetyl CoA and citrate are essential intermediates for lipid synthesis. Thus, GBM tumor cells’ functioning mitochondria reroute Acetyl CoA and citrate to generate lipids. Inhibition of ATP citrate lyase (ACL), an enzyme that catalyzes the conversion of citrate into Acetyl CoA, with hydroxyl citrate, has been shown to hinder GBM cell migration, clonogenicity, and brain invasion in vitro [20]. In addition, targeting key enzymes in fatty acid synthesis and elongation has shown considerable therapeutic potential in GBM xenograft models [21,22].

Although the origin of GBM cells remains unclear, the higher glycolytic capacity of glial cells compared to neurons [23] may explain the GBM tumor cells’ preference towards glycolysis. While glycolysis is less efficient than OXPHOS, it is more efficient at producing ATP, which is preferred, given the taxing demand of GBM tumor cells. However, insufficient evidence suggests that ATP is a limiting factor in highly proliferative cells, as many undergo aerobic glycolysis regardless of nutrient and oxygen availability [24]. In addition to that, recent studies indicate that the choice of metabolic pathway is driven not only by energy production but also by the need for biosynthesis of cellular machinery and adaptation to TME [25]. The “go and grow” hypothesis posits that GBM cells exhibit either proliferative or migrative behavior, but not both simultaneously [26]. This behavior results from metabolic adaptation between two major metabolic pathways: pentose phosphate pathway (PPP) and glycolysis. The PPP, a metabolic pathway parallel to glycolysis, generates ribose-5-phosphate and nicotinamide adenine dinucleotide phosphate (NADPH) from G-6-P for nucleotide synthesis. The dichotomous association of PPP with proliferation and glycolysis with migration is independent of oxygen availability. However, under hypoxic conditions, GBM cells downregulate PPP enzymes, such as glucose-6-phosphate dehydrogenase (G6PD), 6-phosphogluconate dehydrogenase (PGD), and transketolase (TKT) adjuvant, while upregulating glycolysis enzymes like HK2, 6-phosphofructokinase platelet type (PFKP), aldolase C (ALDOC), PKM2, and lactate dehydrogenase A (LDHA). This shift favors increased migration and decreased proliferation [27]. Thus, metabolic pathways are preferentially activated based on oxygen availability; low-oxygen conditions promote migration (or escape a non-conducive environment), while normoxic conditions drive proliferation and growth.

##### Therapeutic Strategies Against Glucose Metabolism Rewiring in GBM

Glucose metabolism in GBM is critical for neoplastic cells to proliferate, migrate, and adapt to the dynamic TME. Therefore, targeting the key components of glycolysis, glucose transporters, and PPP offers promising therapeutic avenues.

GLUT1 inhibitors disrupt glucose transport into tumor cells, thus compromising tumor cell survival. Studies have shown that targeting specific conformational binding sites of GLUT1 at residues such as Phe291 and Trp412 is effective [28]. Natural inhibitors, including resveratrol, ganoderic acid A (GAA) [29,30], and synthetic inhibitors such as Glutor, suppress GLUT1 function, inducing apoptosis [31]. Since GBM tumor cells predominantly rely on aerobic glycolysis, targeting key enzymes, such as HK2 [32] and LDH isoforms (LDHA and LDHB) [33,34], to disrupt glycolytic pathways holds therapeutic potential. As such, potent LDH inhibitors like 5-aminolevulinic Acid (5-ALA) and oxamate, an isosteric form of pyruvate that blocks LDHA activity by competing with pyruvate, abate glycolytic activity and energy production, leading to cell death in glycolysis-dependent GBM cells [35,36]. Furthermore, LDH inhibition has also been shown to sensitize GBM cells towards radiation and chemotherapy, thereby augmenting survival rates. ATP citrate lyase (ACLY) converts citrate into acetyl-CoA, linking glucose and lipid metabolism [37,38], and inhibiting ACLY reduces lipid synthesis, cell proliferation, and tumor growth in GBM [37]. However, challenges remain with small-molecule ACLY inhibitors due to poor cell penetration and affinity [37].

HIF1α regulates genes involved in glucose metabolism, angiogenesis, and tumor cell survival under hypoxic conditions. HIF1α inhibitors, such as echinomycin, attenuate tumor growth and promote apoptosis in hypoxic GBM cells [39]. Additionally, interfering with the HIF1α-platelet-derived growth factor D (PDGFD)-PDGF-receptor α (PDGRα)-AKT impairs the feedforward loop of HIF1α perpetuation in the hypoxic tumor core [39]. In line with this, disruption of the epidermal growth factor receptor (EGFR)-PI3K/AKT pathway reduces GBM cell proliferation, survival, glycolysis dependence, and treatment resistance [40]. Under normoxic conditions, HIF1α undergoes hydroxylation and is degraded via the ubiquitin–proteasome pathway [41]. Therapeutic strategies that mimic normoxic conditions can facilitate HIF1α degradation and restore the TCA cycle, thus reducing the reliance of GBM hypoxic cells on glycolysis, impairing their survival in the oxygen-deprived tumor core [42]. Unfortunately, GBM cells can adapt to HIF1α inhibition by utilizing alternate metabolic pathways, such as creatine metabolism and GLUT14-mediated glucose uptake [43]. Therefore, understanding these adaptations is critical for developing combination therapies. Pyruvate carboxylase (PC) has shown promise in targeting GBM stem cells, especially with low glutamine availability [44]. Alternatively, the combination of PC inhibition and glutamine blockade synergistically inhibits the proliferation of GBM tumor cells [45]. On the other hand, 2-deoxyglucose has been shown to inhibit glycolysis in animal models but aggravate migration and invasion through alternate pathways [46], underscoring the need for combination therapies to target multiple GBM metabolic pathways.

#### 2.1.2. Lipids: Acetate, Cholesterol, Fatty Acids, and Autophagy

^13^C-glucose imaging of GBM patients and patient-derived xenografts of mouse brains revealed that <50% of the carbons of acetyl CoA were of glucose origin [47], suggesting that GBM tumor cells utilize alternate carbon sources to manufacture ATP. One such carbon source is acetate, a short-chain fatty acid with a two-carbon structure that reacts with coenzyme A in the presence of ATP to generate Acetyl CoA through nucleocytosolic acetyl-CoA synthetase (ACSS2) enzymatic reactions [48]. This adaptation in oxidizing acetate in the citric acid cycle ensures that high biosynthetic and bioenergetic demands for malignant tumor growth are met. Interestingly, >50% of the acetyl CoA carbon pool in GBM cells is derived from acetate, while in healthy brain cells, <10% of acetyl CoA comes from acetate [47].

The brain is a cholesterol-rich organ, containing 20% of the body’s total cholesterol, which is locally synthesized by astrocytes [49] since lipoprotein-linked peripheral cholesterol cannot efficiently cross the BBB. It is important to note that GBM tumor cells take up significantly more cholesterol than the surrounding non-tumor cells, and their survival depends on it. A recent study has shown that the liver X receptor (LXR) agonist LXR-623 is selectively effective against GBM cells in an LXR-β and cholesterol-dependent manner, engendering tumor regression and prolonged survival in patient-derived GBM xenograft mouse models [50].

On the other hand, the autophagy pathway, known to provide therapeutic resistance for GBM cells, is initiated by a series of active proteins including autophagy-related (ATG) proteins, ultraviolet radiation resistance-associated gene (UVRAG), beclin 1, phosphoinositide 3-kinase catalytic subunit type 3 (PIK3C3), microtubule-associated protein 1A/1B-light chain 3 (LC3), and p62 [51]. These proteins work together to facilitate the formation of autophagosomes, where cellular components are delivered to the lysosomes for degradation, releasing free fatty acids. Unlike cholesterol, free fatty acids can cross the BBB and are converted into Acetyl CoA in the mitochondria, promoting the citric acid cycle to produce ATP, also known as β-oxidation. A ^13^C-Octanoate imaging study showed that this medium-chain fatty acid constitutes almost 13% of humans’ normal free fatty acid pool, providing approximately 20% of the total brain oxidative energy production [52]. However, the mechanism governing the transport of fatty acids into the mitochondria remains unclear and warrants further investigation.

##### Therapeutic Strategies Against Lipid Metabolism in GBM

GBM is caused by altered lipid metabolism, which plays a crucial role in tumor growth and survival. As a result, researchers are keen to target aberrant lipid metabolism in tumor cells—including de novo lipogenesis, fatty acid oxidation, and lipid uptake—since these pathways hold significant therapeutic potential in both preclinical and clinical settings.

The stearoyl CoA desaturase (SCD) inhibitor YTX-7739 induces lipotoxicity in patient-derived GSCs and has shown therapeutic efficacy in GBM mouse models. Although it is pathway-specific, mitogen-activated protein kinase kinase (MEK)—extracellular signal-regulated kinase (ERK) signaling increases sensitivity towards SCD inhibition, whilst the activation of adenosine monophosphate-activated protein kinase (AMPK) aggravates treatment resistance [53]. Furthermore, inhibiting triglyceride synthesis abates lipid droplet accumulation and reduces cell proliferation [54]. GBM tumor cells preferentially upregulate diacylglycerol-acyltransferase 1 (DGAT1) to store excess fatty acids as triglycerides and lipid droplets, thereby avoiding lipotoxicity. Inhibiting DGAT1 in xenograft models disrupts lipid homeostasis, forcing excessive fatty acids into the mitochondria, which elicits high levels of reactive oxygen species (ROS), leading to mitochondrial damage, cytochrome c release, and apoptosis [55]. Another study demonstrated that fatostatin treatment effectively downregulates AKT phosphorylation, leading to apoptosis and cell cycle arrest in various GBM-derived tumor cell lines [56]. The reliance of GBM tumor cells on lipid metabolic reprogramming poses a promising strategy to exploit the metabolic vulnerabilities of these tumor-initiating cells. Further research and clinical trials are warranted to validate these approaches and translate them into effective therapies for GBM patients.

#### 2.1.3. Amino Acid: Glutamine-Derived Precursors and Tryptophan

Glutamine, the most abundant amino acid in the human body, is a nitrogen and carbon source for the biosynthesis of nucleotides and amino acids for GBM growth [57]. Isotopic tracing studies using both GBM cell line-derived and patient-derived xenografts in mice have shown that glutamine-derived precursors are not the major contributors to the TCA cycle [47,58]. However, glutamine can be metabolized to α-ketoglutarate via two main pathways: 1. glutaminase catalyzed the hydrolysis of glutamine to glutamate and ammonia, followed by conversion of glutamate to α-ketoglutarate catalyzed by glutamate dehydrogenase or by a glutamate-linked aminotransferase (transaminase); and 2. by the glutaminase II pathway, where glutamine transaminase is coupled to ω-amidase to form the corresponding α-keto acid, which is then hydrolyzed to α-ketoglutarate and ammonia [59]. The resulting α-ketoglutarate can enter the TCA cycle in the mitochondria, creating an electrochemical gradient required for ATP production. Glutathione biosynthesis, which is dependent on glutamine metabolism, has been shown to confer resistance to oxidative stress and inflammation [60], a feature common in cancer. While specific evidence for GBM neoplastic cells is lacking, glutamine utilization is significantly augmented in GBM tumor cells.

Recent reports have shown that GBM patients exhibit decreased serum levels of tryptophan compared to healthy individuals. Additionally, downstream metabolites of tryptophan catabolism were also reduced, with these changes more pronounced in the early stages of the process, suggesting that tryptophan availability regulates the levels of its metabolites. Moreover, the concentration of the systemic metabolites, including kynurenine, directly correlated with the GBM tumor mass [61]. Kynurenine, synthesized via the kynurenine pathways and catalyzed by tryptophan dioxygenase, plays an immunosuppressive role in GBM tumor formation and progression [62]. Interestingly, genes involved in tryptophan metabolism, such as aryl hydrocarbon receptors that are associated with poor overall survival in GBM patients, were expressed in almost all cell types, particularly T cells [61].

Arginine is rapidly metabolized by activated T cells, significantly reducing intercellular arginine levels. Many studies have reported that arginine deficiency can hinder T cell effector function, increase the production of immunosuppressive molecules, and impair the proliferation of chimeric antigen receptor T cells (CAR-T), thus limiting therapeutic effects [63,64]. Arginine metabolism is primarily mediated by the enzyme arginase, which converts arginine into urea and ornithine. In GBM, both arginine transporters and arginase are upregulated since tumor cells take more arginine from the TME to fuel bioenergetics and biosynthesis pathways [65]. This leads to local and systemic arginine deficiency, as well as the accumulation of arginine metabolism byproducts. Bone marrow-derived monocytic cells (M-MDSC) with augmented arginase I (Arg1) expression have been shown to exhibit functional similarities to M2-like macrophage phenotypes, releasing anti-inflammatory cytokines including interleukin (IL)-10, transforming growth factor β (TGFβ), and indoleamine 2,3-dioxygenase (IDO), an enzyme involved in tryptophan metabolism [66,67]. Thus, it engenders a downregulation of the Cluster of Differentiation (CD) 3ζ, hindering CD4+ and CD8+ T cells’ effector function and promoting immunosuppressive phenotypes [68].

##### Therapeutic Strategies Against Amino Acid Metabolism

Recent studies have explored glutamine inhibition strategies to target glutamine-addicted GBM tumor cells. For instance, one study demonstrated the significant potential of glutamine-starvation therapy combined with photo-enhanced hemodynamic therapy in disrupting glutamine metabolism and increasing ROS, which augmented tumorigenic GBM cell death [69]. In another approach, the inhibition of glutamine metabolism via Epigallocatechin-3-gallate (EGCG) hindered GBM tumor growth in both GBM cell lines and patient-derived mouse xenograft model settings [70]. Additionally, a pharmacological intervention targeting glutaminase with the inhibitor dibenzo[*b*,*f*][1,5]dioxocin to curtail glutaminolysis demonstrated anti-migratory and anti-proliferative properties in GBM cell lines [71]. In a separate study, the glutamine antagonist 6-diazo-5-oxo-L-norleucine (DON), in combination with a calorie-deficit ketogenic diet, was administered to syngeneic GBM mouse models. This synergistic diet/drug therapeutic strategy increased GBM cell death while mitigating edema, hemorrhage, and inflammation, ultimately improving survival rates [72].

Tryptophan metabolism, on the other hand, can be targeted by inhibiting IDO1 enzymatic activity utilizing a novel inhibitor, GDC-0919. The outcome of the study indicated the potential to enhance the efficacy of radiation therapy by activating immune-related responses [73]. Alhough few studies have focused on arginine metabolism, this metabolic reprogramming can influence tumor growth and immune responses in GBM. Therefore, arginine deprivation or inhibiting its metabolic pathways could hinder GBM tumor proliferation and disrupt the TME, making it a promising target for therapeutic interventions. Further research in this area will be highly beneficial, especially regarding multi-modal therapies.

### 2.2. Genetic Modifications and Metabolic Rewiring

#### 2.2.1. *Isocitrate Dehydrogenase* (*IDH*) Mutations

*IDH* mutations, one of the key genetic alterations in GBM, lead to the production of 2-hydroxyglutarate (2HG), which inhibits α-ketoglutarate-dependent dioxygenases. Thus, 2HG affects various cellular functions, including redox balance and energy production. The accumulation of 2HG also alters HIF and glycolysis pathways, leading to changes in cellular metabolism and epigenetic modifications [74,75]. In the hypoxic core of the tumor, 2HG accumulation drives the shift toward glycolysis and stabilizes the expression of HIF1α, further upregulating genes involved in glycolysis, angiogenesis, and pH regulation [76]. At the tumor’s leading edge, where nutrients and oxygen are more readily available, tumor cells are more proliferative, and *IDH* mutations also promote stem-like phenotypes [77,78]. Altered metabolism enhances resistance to therapies by supporting anabolic processes [78]. In the infiltrating zone, metabolic adaptations are critical for tumor cell invasion, survival, and expansion, allowing the tumor cells to evade immune surveillance [79]. Tumor cells increasingly rely on alternate carbon sources, and elevated 2HG production due to *IDH* mutations modulates tumor-associated macrophages (TAMs) and other immune cells, fostering an immunosuppressive microenvironment [76].

#### 2.2.2. *Epidermal Growth Factor Receptor* (*EGFR*) Amplifications

*EGFR* amplifications promote glycolysis and lipid synthesis to support high-energy biosynthetic demands by enhancing glycolytic enzymes and promoting glucose uptake [80]. This is also linked to increased glutamine dependency, further improving metabolic adaptability for GBM tumor cells [81]. In hypoxic conditions, EGFR signaling activates the PI3K/AKT pathway, supporting cell survival and proliferation in low oxygen levels [82]. At the tumor’s leading edge, EGFR promotes lipid metabolism and fatty acid synthesis to support membrane biogenesis and cell proliferation [83]. Additionally, the EGFR/AKT pathway activates the mevalonate pathway, increasing cholesterol and other isoprenoid synthesis, providing metabolic flexibility for GBM cells to adapt to neighboring microenvironments and sustain their invasive capabilities [80,83]. In the infiltrating zone, *EGFRvIII* mutations activate the Src family kinase, c-Src, which supports angiogenesis. The increased oxidative stress and lipid peroxidation generate highly invasive cells with elevated aldehyde dehydrogenase (ALDH) [82,83]. Thus, *EGFR* amplifications not only promote the invasiveness of GBM tumor cells but also contribute to their resistance to EGFR-targeted therapies.

#### 2.2.3. *Phosphatase and Tensin Homolog* (*PTEN*) Loss

Loss of the tumor suppressor gene, *PTEN*, is associated with mitochondrial dysfunction, leading to increased reliance on glycolysis to support tumor growth [77,84]. Under hypoxic conditions, *PTEN* loss contributes to the accumulation of lipid droplets, which are essential for membrane synthesis and energy storage [85]. At the tumor’s leading edge, *PTEN* loss increases Ras homolog family member B (RHOB) expression, augmenting cell motility and adhesion [86]. Additionally, enhanced glutamine metabolism observed in *PTEN*-deficient GBM cells supports nucleotide synthesis and maintains redox balance, enabling robust and sustained proliferative rates [77,87]. In the tumor’s infiltrative zones, *PTEN*-deficient GBM cells secrete increased levels of C-C Motif Chemokine Ligand 2 (CCL2), facilitating the recruitment of myeloid cells, thereby promoting tumor growth and suppressing immune activity [88]. Cancer stem cells in *PTEN*-deficient tumors also display distinct lipid profiles characterized by lower lipid droplet accumulation but increased production of polyunsaturated fatty acids. This metabolic shift helps maintain stem cell properties and promotes tumorigenesis [84,85].

#### 2.2.4. Other Genetic Mutations: O-6-Methylguanine-DNA Methyltransferase (MGMT) and Tumor Protein 53 (TP53)

In addition to *IDH*, *EGFR*, and *PTEN* alterations, several other genetic mutations contribute to the metabolic rewiring in GBM TME. Methylation of *MGMT* results in the accumulation of DNA damage, inducing metabolic stress in GBM tumor cells. To survive, these cells switch to glycolysis to generate sufficient ATP, which is associated with the aggressive growth of GBM and its resistance to therapy [77]. In its functional state, p53 reduces glycolytic flux by downregulating GLUT1/4, GLUT3, and HK2, favoring OXPHOS. When *TP53* is mutated, this regulatory effect is lost, redirecting metabolism towards glycolysis. Additionally, mutant p53 proteins can acquire new functions that vary depending on the specific mutants, affecting glycolytic and mitochondrial pathways differently [89,90].

#### 2.2.5. Therapeutic Strategies for Genetic Modification-Induced Metabolic Rewiring

Although *IDH* mutations in GBM are generally associated with a favorable prognosis, especially in younger patients, recent findings showcase specific metabolic dependencies associated with *IDH* mutations. The production of the oncometabolite 2HG [77], which can be targeted by specific inhibitors, suggests that targeting *IDH* mutations may improve therapeutic outcomes in GBM.

Despite the availability of EGFR tyrosine kinase inhibitors (TKIs), clinical trials have shown limited success due to the malignant adaptive resistance mechanisms employed by GBM tumor cells. However, recent studies have proposed a dual kinase inhibition strategy—targeting cyclin-dependent kinase 6 (Cdk6) and discoidin domain receptor 2 (Ddr2)—to simultaneously target both EGFR and compensatory pathways activated by EGFR inhibition. This approach may overcome acquired resistance and enhance the efficacy of EGFR-targeted therapies [91,92]. Moreover, combining epigenome therapies with EGFR inhibitors has shown promise in preclinical animal models [93]. *EGFR*- and *PTEN*-induced mutations generate distinct carbamoyl-phosphate synthetase 2, aspartate transcarbamylase, and dihydroorotase (CAD) phosphorylations, which activate carbon influx via pyrimidine synthesis. Therefore, inhibiting these specific driver mutations can mitigate pyrimidine synthesis and GSC tumorigenic capacity in vitro [94]. Furthermore, cinobufagin, a traditional Chinese medicine-derived cardenolide compound, has shown selective anti-cancer effects in tumors with *EGFR* amplifications and *PTEN* deletions, warranting further studies to validate its efficacy [95].

## 3. Immune Infiltration in GBM TME

A strong association exists between hypoxia, metabolic remodeling, and immune dysregulation across various diseases. In GBM, hypoxia-induced mitochondrial-derived ROS destabilize HIFα, which triggers metabolic reprogramming to sustain bioenergetics and biosynthesis processes in the hypoxic TME. Likewise, while quiescent immune cells are metabolically inactive, their activation during inflammation requires significant metabolic shifts to produce ATP for effector function. This process relies heavily on the HIF pathway, which acts as the critical transcriptional regulator of immunity and inflammation [96]. This complex interplay between hypoxia, metabolic remodeling, and immune dysregulation shapes the immune cells’ function and orchestrates their recruitment and differentiation within the GBM TME. Hypoxia-induced signals and tumor-derived factors collaboratively establish a milieu that attracts peripheral monocytes and reprograms resident immune cells, driving them toward immunosuppressive phenotypes.

GBM cells secrete Cysteine-rich 61 (CCN1) to recruit monocytes from the bloodstream into the TME [97]. Within the TME, these monocytes differentiate into macrophages and are driven to adopt an anti-inflammatory phenotype under the influence of chemokines, cytokines, and hypoxic signals [98]. TAMs play a key role in immune suppression and tumor progression in GBM [99]. Microglia, the brain’s resident immune cells, also contribute to tumor growth and immune evasion. These microglia infiltrate the GBM TME via lysyl oxidase (LOX) and olfactomedin like-3 (OLFML3) signaling [100], adopting an immunosuppressive phenotype driven by pathways such as mTOR, STAT3, and NF-κB that hinders T cell infiltration, thereby facilitating immune evasion [101].

T cell recruitment into the GBM TME is primarily driven by chemokine such as C-X-C motif chemokine ligand (CXCL) 10, which attract CD8+ T cell and effector memory T-cells, to enhance the efficacy of immunotherapies and improve therapeutic outcomes [102]. However, chronic exposure to tumor antigens and the immunosuppressive TME often drives these T cells to exhaustion, limiting their anti-tumor activity [103,104]. By contrast, regulatory T cells (Tregs) are preferentially recruited by GBM through the secretion of chemokines such as C-C motif chemokine ligand (CCL) 2 and CCL22 [105]. Once in the TME, Tregs amplify immunosuppression by releasing inhibitory cytokines, disrupting metabolic pathways, and suppressing the function of effector T cells [106,107,108].

Similarly, myeloid-derived suppressor cells (MDSCs) play a critical role in the immunosuppressive TME of GBM by impairing the function of CD4+ T cells [109]. GBM cells release chemokines such as CCL2 and CCL7, which recruit MDSCs through chemokine receptors like C-C chemokine receptor type 2 (CCR2) and C-X3-C chemokine receptor 1 (CX3CR1) [110]. Within the TME, MDSCs share metabolic pathways with GBM cells, supporting their survival and exacerbating T-cell dysfunction [111].

### 3.1. Immunoregulatory Factors in GBM TME

**Figure 2:**
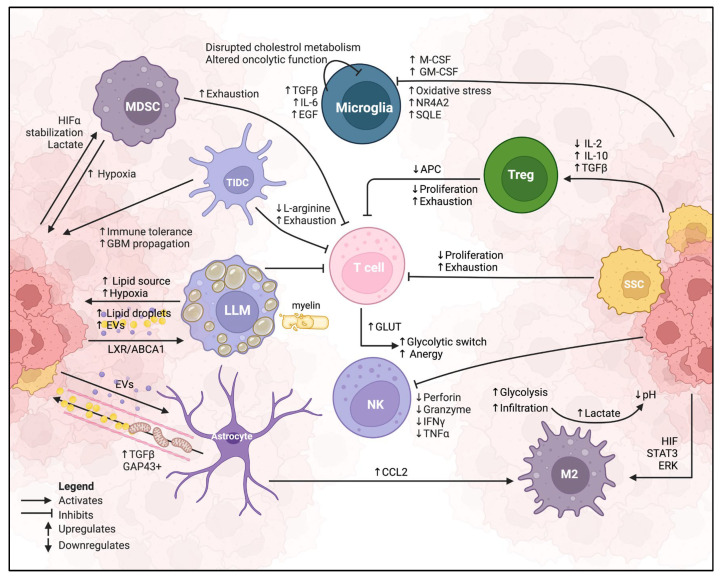
Schematic representation of the GBM TME infiltrated by various immune cells. GBM neoplastic cells release immunosuppressive signals via metabolic and immunological reprogramming, driving the immunosuppressive phenotype. Prolonged antigen exposure exacerbates T cell exhaustion, impairing metabolic activity and promoting the glycolytic switch and anergy. Conversely, Tregs thrive in these malignant conditions, further suppressing T cells’ effector dysfunctions. Through lactate signaling, GBM polarizes macrophages to adopt the M2-like phenotype (M2), fostering immune evasion. SCCs and LLM promote lipid-driven tumorigenic cascades to promote T cell exhaustion further. Tumor-associated astrocytes (TAAs) exposed to EVs from the GBM TME support the growth and progression of GBM cells by transferring mitochondria and cholesterol metabolism through contact-dependent tubules. These highly complex metabolic-induced GBM malignant cell, TME, and immune cell crosstalks establish a destructive feedback loop that promotes GBM growth and immune evasion.

The GBM TME is enriched with immunoregulatory factors that attract and exploit immune cells to support tumor progression (Figure 2). Hepatocyte growth factor (HGF) and its receptor cellular mesenchymal–epithelial transition factor (c-Met), expressed on both macrophages and GBM cells, promote microglia and peripheral macrophage infiltration [112]. Monocyte chemotactic protein 3 (MCP3) also plays a crucial role in immune cell chemotaxis, guiding macrophages, monocytes, natural killer (NK) cells, T cells, and dendritic cells into the TME by binding to CCR-2 and CCR-3 receptors [113]. In addition to that, glial-derived neurotrophic factor (GDNF), expressed by both neurons and glial cells in GBM, promotes microglia infiltration and enhances GBM cell migration [114]. Once infiltrated, immune cells are polarized by GBM-secreted factors, such as TGF-*β*2, into the immunosuppressive M2-like phenotype, promoting tumor growth and survival [115,116]. Periostin, an ECM protein downstream of TGF-β2 [117], further polarizes macrophages and enhances tumor malignancy. Knockdown of periostin in rodent xenograft models has been shown to reduce M2-like macrophage polarization and impede GBM growth [118]. To enhance their migratory and invasive phenotypes, GBM remodels the ECM by secreting cadherin and osteopontin [119] and increases IL-8 expression to promote F-actin polymerization that is EMT-like [120,121]. Importantly, blocking IL-8 receptor binding reduces GBM migration and invasion [122,123].

Neoangiogenesis, another key aspect of ECM remodeling, is driven by inflammatory mediators such as cyclooxygenase-2 (COX2), TGF-β, IL-8, and IL-6 [124,125,126]. COX2 catalyzes the production of eicosanoids that enhance GBM angiogenesis, growth, immune evasion, and suppression downstream via the synthesis of vascular endothelial growth factor (VEGF) [125,127]. Furthermore, VEGF is upregulated by TGF-β via activation of Ras protein and the mitogen-activated protein kinase (MAPK) pathway [128,129].

GBM tumor cells also modulate the TME to promote an anti-inflammatory response by downregulating pro-inflammatory cytokines such as TNFα and TNF-related apoptosis-inducing ligand (TRAIL), inhibiting apoptosis and immune response [130]. By contrast, IL10 levels are elevated in GBM [131], promoting tumorigenesis by suppressing CD8+ T cell cytotoxicity, phagocytosis, and activating Tregs [132,133]. Additionally, the B7-homolog (B7-H1), an immunosuppressive protein expressed on GBM cells, binds Programmed Cell Death Protein 1 (PD-1) to impair T helper cell immune response and prevent apoptosis, aiding immune evasion [134,135].

### 3.2. Metabolic Rewiring and Its Impact on the Immune Landscape of the GBM TME

A myriad of immune cells infiltrate the GBM TME, including CD4+ T helper, CD8+ T cytotoxic, and Tregs [136]. Despite this immune infiltration, GBM cells establish an immunosuppressive TME that drives immune cells into exhaustion, characterized by the expression of inhibitory co-receptor T cell immunoglobulin and mucin-domain-containing 3 (TIM-3) and PD-1 [137,138]. This exhaustion impairs T cell function [137] and immune memory [138]. While CD4+ T cells secrete interferon-gamma (IFN*γ*), a pro-inflammatory cytokine, to recruit more T cells, CD8+ T cells often become dysfunctional due to prolonged exposure to tumor antigens [139]. These exhausted T cells compete with GBM cells for essential nutrients in the hypoxic microenvironment. The nutrient competition, coupled with aggressive metabolic demands of GBM, shift T cell metabolism from OXPHOS to glycolysis via the mTOR pathway, further impairing T cell activity and promoting tumor progression [140]. The increased dependency on glycolysis generates an acidic TME, which is exacerbated by hypoxia and lactate accumulation in a feedforward loop fashion, further impairing cytolytic T cell activity and inducing anergy [141].

The high-lactate, low-glucose TME favors immunosuppressive immune cells such as Tregs and MDSCs. Tregs suppress cytotoxic T lymphocytes (CTL), and CD4+ helper T cells function by depleting IL-2 and upregulating IL-10 and TGF-β [142]. Tregs also impair antigen-presenting cell (APC) function and reduce cytokine-producing T cell proliferation [143,144]. Their elevated presence correlates with higher glioma grades and worse prognosis [145]. MDSC exploits the lactate-induced stabilization of HIF*α* to suppress T cell activities [146], exacerbate hypoxic conditions [147], and perpetuate the acidification of the GBM TME, creating a persistent immunosuppressive environment [148]. Lactate production in the glycolytic TME also reduces NK cell cytotoxicity by limiting perforin and granzyme release and inhibiting the production of IFN*γ* and TNF*α* [147,149,150].

The lactate-rich microenvironment drives M2-like polarization of macrophages through signaling pathways such as ERK, STAT3 [151], and HIF1*α* [152]. Lactate induces lactylation, a post-translational modification of histone proteins by lactyl groups [153], further promoting the pro-tumor phenotype of macrophages. Additionally, high lactate levels inhibit monocyte differentiation into dendritic cells [154], limiting the tumor’s ability to mount an effective immune response. The acidic TME, coupled with macrophage infiltration, augments the expression of sodium/hydrogen exchanger 1 (NHE1), promoting glycolytic metabolism and perpetuating the acidic environment [155]. This creates a feedback loop between TAMs and GBM cells that fosters Treg accumulation and further M2 polarization, reinforcing an immunosuppressive milieu [156].

In parallel, slow-cycling GBM cells (SCCs) contribute to the immunosuppressive TME by recruiting pro-tumorigenic macrophages and myeloid cells, impairing T cell-mediated anti-tumor activity [157,158]. Furthermore, SCCs, which preferentially rely on lipid metabolism, reprogram T cell metabolism from glycolysis to fatty acid oxidation, a shift associated with T cell senescence and diminished cytolytic activity [159].

A key feature of the GBM TME is the accumulation of lipid-loaded tumor-associated foam cells (TAFs) in the peri-necrotic niche [160,161]. These TAFs acquire high levels of lipid droplets by engulfing necrotic debris and tumor cell-derived extracellular vesicles (EVs), augmenting the hypoxic response and increasing VEGF, as well as HGF secretion. This, in turn, contributes to immune suppression by recruiting additional pro-tumor macrophages and inhibiting T cell-mediated anti-tumor activity [160,162]. Additionally, microglia in the GBM TME experience significant oxidative stress that triggers the activations of nuclear receptor subfamily 4 group A member 2 (NR4A2) and squalene monooxygenase (SQLE), disrupting cholesterol metabolism [163], impairing antigen presentation, and contributing to an immunosuppressive, tumor-promoting environment [164,165]. A subpopulation of glycoprotein nonmetastatic melanoma protein B^high^ lipid-laden microglia and macrophages (LLMs) was recently identified in the TME of genetically modified GBM mouse models. These cells recycle myelin, thus providing lipids as an energy source to cancer cells, further sustaining the mesenchymal phenotype of GBM cells via the Liver X receptor/ATP-Binding Cassette Subfamily A Member 1 (ABCA1)-dependent pathway [166].

Tumor-infiltrating dendritic cells (TIDCs) play a crucial role in impeding T cell immune surveillance in the GBM TME, contributing to tumor progression [167]. T cell immunoreceptors with IG and ITIM domains (TIGIT), which are expressed on T cells, bind with high affinity to the poliovirus receptor on TIDCs, inhibiting T cell activation in vitro and increasing interleukin production by dendritic cells [168]. The augmented PD-1 expression on TIDCs further blocks co-stimulatory signals, preventing T-cell activation [169]. Immature dendritic cells also contribute to immune tolerance, suppressing antigen-specific T cell immune activation and increasing antigen-specific IL-10 secretion by CD8^+^ T cells following a single injection of antigen-pulsed dendritic cells [170]. Furthermore, MDSC produces ROS and other immunosuppressive cytokines that promote the expansion of Tregs, further inhibiting CD8^+^ cell activation [171]. The upregulation of PD-1 expression on T cells also leads to their exhaustion, overwhelming the CD4+ T cell response [109] TIDC immunosuppression is primarily due to L-arginine metabolism by arginase, depleting L-arginine levels in the GBM TME and impairing T-cell functions [172].

Astrocytes in the GBM TME play a crucial role in immune and metabolic modulation. TAAs recruit TAMs into the TME and reprogram them into pro-tumorigenic phenotypes through cytokines such as CCL2, contributing to an immunosuppressive TME [173]. TAAs also contribute to the metabolic landscape by supplying cholesterol to GBM, supporting their survival. The depletion of reactive astrocytes has been shown to halt GBM progression and prolong survival in experimental models [173]. GBM malignant cells are thought to induce metabolic shifts on astrocytes via EV-mediated transfer of full-length mRNAs, supporting the oncogenic transformation of astrocytes and fostering GBM tumor progression [174]. Transcriptomics analysis revealed that tumor-conditioned astrocytes were enriched with genes associated with metabolic reprogramming, such as those involved in Warburg effects and brain tumors [174]. Recent studies showed that TAAs also transfer mitochondria to GBM cells via growth-associated protein 43 (GAP43)-positive tumor microtube-like structures [175], and TGFβ [176], enhancing ATP production and promoting tumor growth, aggressiveness, and therapy resistance [177,178].

Metabolic rewiring in GBM reshapes the immune landscape, with interactions between GBM cells and immune cells creating an environment that both sustains tumor growth and promotes immune evasion. This rewiring varies across GBM subtypes—classical, mesenchymal, and proneural. The mesenchymal subtype exhibits the most pronounced metabolic alterations, including an abundance of L-fucose and core fucosylation [179], which set it apart from both the classical and proneural subtypes. By contrast, the proneural subtype displays the least degree of metabolic rewiring, highlighting the inherent metabolic heterogeneity among these subtypes. Both mesenchymal and classical subtypes exhibit upregulation of immune–metabolic axes, like tryptophan, arginine, and adenosine metabolism, contributing to the accumulation of Tregs and M2-like macrophages [180]. Notably, the mesenchymal subtype is marked by the highest levels of immune cell infiltration, including TAMs, microglia, CD8+ T cells, CD3+ T cells, and FOXP3+ Tregs, compared to the classical and proneural subtypes [181,182].

Given these distinct metabolic and immune features, subtype-specific targeted therapies that disrupt tumor-induced metabolic reprogramming hold great promise for restoring immune defenses, enhancing anti-tumor responses, and ultimately improving therapeutic outcomes.

### 3.3. Therapeutic Potential Against Immune Exploitation in the GBM TME

The exploitation of immune cells by GBM neoplastic cells, coupled with the metabolic constraints imposed by the TME, have rendered immune cells ineffective in abrogating tumor growth and invasion. Consequently, recent therapeutic strategies have focused on restoring immune functionality, with significant advancements centered on reshaping the metabolic landscape of immune cells to enhance their anti-tumor activity (Table 1).

Although adoptive cell therapies (ACTs) show promising results in preclinical settings, their efficacy in the clinical setting remains limited, partly due to the metabolic-induced anti-tumor suppression of the cytolytic cells. One study proposed maintaining T cells in a less differentiated state during ex vivo through lipid-induced mitochondrial rewiring to ensure a more adaptable metabolism, thereby overcoming the malignant metabolic rewiring in the GBM TME [183]. mTOR signaling plays a crucial part in the metabolic rewiring of T cells from OXPHOS to glycolysis. Targeting mTOR pathways with rapamycin and its analogs, such as Apitolisib (GDC-0980) and NVP-BEZ235, can modulate GBM TME and improve T cell functionality by inhibiting mTORC1 [185,203]. However, the efficacy of this monotherapy has been limited, given that rapamycin only inhibits a subset of the Mechanistic Target of Rapamycin Complex 1 (mTORC1) substrates [184]. mTORC1 enhances glucose flux through the pentose phosphate pathway (PPP) and redirects it back into glycolysis, bypassing glycolytic blockages. This underscores the synergistic relationship between mTORC signaling and glycolysis-driven metabolic addiction in GBM. Combining mTOR and glycolytic inhibitors to target the mTOR-facilitated glycolytic switch in T cell metabolism may improve therapeutic efficacy [204].

Another modality to enhance T cell function involves improving mitochondrial fitness and OXPHOS through metabolic adjuvants. Peroxisome Proliferator-Activated Receptor Gamma Coactivator 1-Alpha (PGC-1α), a key regulator of OXPHOS and fatty acid oxidation, can be used to promote mitochondrial biogenesis and restore T cell functions. Indeed, an inhibition-resistant, engineered PGC-1α can metabolically reprogram CAR-T cells to stimulate mitochondrial biogenesis, maintain metabolic fitness, and restore effector functions [205]. In addition to that, while CD47 is better known for immune evasion, recent studies suggest its role in the malignant metabolic switch towards glycolysis in the GBM TME, suggesting that blocking CD47 may enhance microglia-mediated cytotoxicity against GBM [186].

Neutralizing immunosuppressive cytokines, such as TGF-β and IL-10, offers another strategy to reprogram the TME, potentially reversing its immunosuppressive feature and reinstating T cell effector functions. Strategies like bispecific CAR-T and targeted delivery of IL13RA2/TGF-β show promise in enhancing immune responses and improving therapeutic outcomes [187,206]. IL-15 is crucial for NK cell function, and augmenting the IL-15 signal by inhibiting the negative regulator—Cytokine Inducible SH2-Containing Protein (CIS)—boosts NK metabolic plasticity [207]. Additionally, upregulating amino acid transporters, such as solute carrier family (SLC)1A5, SLC7A5, and SLC3A2, [208] improve nutrient uptake in the nutrient-deprived TME, counteracting malignant metabolic reprogramming and restoring anti-tumor functionality of NK cells.

On the other hand, oncogenic Tregs thrive in low-glucose, high-lactate environments regulated by FOXP3 and mTORC signaling. Disrupting these pathways, for example, using antisense oligonucleotides or deleting mTORC adaptor genes, can induce metabolic impairment in Tregs [188,209]. Additionally, targeting Tregs’ reliance on glycolysis is another promising approach to counteract immunosuppressive functions. For instance, inhibiting glucose uptake and glycolysis in Tregs through TLR8-mediated reprogramming can impair their pro-tumorigenic phenotypes and enhance anti-tumor immunity [189].

HIF-1α plays a central role in the metabolic reprogramming of GBM cells and TAMs under hypoxic conditions. Hence, therapeutic strategies that target HIF-1α stabilization will disrupt the hypoxic TME, reducing its contribution to immune suppression and macrophage polarization. One promising approach involves the development of innovative nanoplatforms, such as macrophage–cancer hybrid membranes, which can specifically target GBM malignant cells and effectively silence HIF-1α under hypoxic conditions [190].

Additionally, targeting lipid metabolism adopted by TAMs to sustain the immunosuppressive TME and promote tumor growth is another promising target for therapeutic intervention. Hence, approaches that inhibit lipid droplet formation [161], target fatty acid oxidation [199], and disrupt cholesterol metabolism [197] have demonstrated encouraging outcomes in various other cancer models. These strategies hold significant potential for application in TAMs within the GBM microenvironment.

Moreover, targeting the SQLE, which regulates the cholesterol metabolism of TAMs, presents a viable avenue to impair the TME. Genetic targeting of NR4A2, including heterozygous and microglia-specific, has been shown to reshape microglia plasticity and enhance the antigen-presenting capacities of CD8^+^ T cells [163]. Similarly, pharmacological inhibition of NR42A using Bay-11-7082 or SQLE using terbinafine attenuates the immunosuppressive TME and improves the efficacy of immune checkpoint blockade therapies [163].

Furthermore, therapeutic strategies targeting TIDCs in GBM can focus on modulating L-arginine metabolism. These approaches include selectively supplementing L-arginine to increase its availability for immune cells while depriving GBM cells of this critical resource [198]. Improved delivery systems, such as brain-targeted liposomal honokiol and the disulfiram/copper codelivery system (CDX-LIPO), have shown potential in modulating the TME immune landscape [200]. Likewise, dendritic-cell-based immunotherapy can be employed to enhance the activation and function of dendritic cells, thereby promoting an effective and more robust anti-tumor immune response [201,210].

Recent findings have demonstrated the proximity-linked crosstalk between astrocytes and GBM neoplastic cells via EVs and tumor microtube-like structures [202]. Understanding the molecular mechanisms behind EV-mediated astrocytic transformation and mitochondrial transfer is crucial, as it may unveil therapeutic opportunities targeting this understudied interaction within the TME. Notably, one study demonstrated that inhibiting the cholesterol efflux from TAAs significantly promotes tumor regression and prolongs the survival of experimental mice in vivo [173], providing a promising avenue for intervention.

Targeting lactate production and the resultant acidification of the GBM TME is a promising therapeutic avenue to disrupt the pro-tumor, immune-suppressive niche. A recent study explored the use of directed deliveries incorporating lonidamine and syrosingopine to target the glycolytic metabolic pathway and the lactate transporter monocarboxylate transporter 4 (MCT4), effectively inhibiting lactate efflux in GBM cells [192]. This intervention led to the neutralization of the TME pH, shifted TAMs to an anti-inflammatory phenotype, increased NK cell numbers, reduced Tregs, and improved immune response in both in vitro and in vivo models [192,211,212]. Other studies have mimicked the LDH catalytic activity to degrade lactate to pyruvate using nanosheets [193,194], encapsulated catalysts [196], and ribonucleic acid interference (RNAi) nanoparticles [195]. These approaches not only reduced the lactate levels within the GBM TME but also activated T cells’ immune response and reprogrammed TAMs to a pro-inflammatory M1-like state, alleviating the immunosuppressive GBM TME. These strategies collectively aim to modulate lactate levels in the TME by targeting lactate metabolism to enhance immune cell function and improve the efficacy of immunotherapies.

In essence, GBM neoplastic cells modulate the TME, driving metabolic rewiring of immune cells. This metabolic alteration leads to immune cell exhaustion and phenotypic change, ultimately impairing their function. Consequently, GBM tumor cells evade immune surveillance and recruit these dysfunctional immune cells to support tumor growth and progression. However, therapeutic strategies should focus on remodeling the malignant metabolic landscape to restore anti-tumorigenic immune activity. Thus, by targeting key metabolic pathways and enhancing immune cell functions, these therapies can disrupt the pro-tumor immunosuppressive niche, thereby improving the efficacy of immunotherapies and ultimately leading to better clinical outcomes.

## 4. Future Perspectives

GBM is characterized by extensive metabolic rewiring, genetic heterogeneity, and an immunosuppressive TME, contributing to its complexity and malignancy. Despite extensive research, effective therapeutic interventions remain limited, underscoring the urgent need for novel approaches.

GBM tumor cells, despite their high energy demands, predominantly rely on aerobic glycolysis. This metabolic strategy allows for flexibility in balancing energy production, prioritizing biosynthesis, and adaptation to an acidic and nutrient-scarce TME. Within the TME, particularly at the invasive front and hypoxic core, GBM tumors dynamically shift between PPP and glycolysis depending on oxygen availability and cellular activity, such as proliferation or migration. Thus, understanding the plasticity that underlies this metabolic adaptability is essential, particularly in the hypoxic regions where GBM faces nutrient stress. Key signaling pathways, including those involving HIF1α and lactylation of histones, require deeper exploration to understand how they affect the activity of immune cell subsets and contribute to immune suppression. Additionally, nutrient competition between GBM tumor cells and immune cells in the TME further compromises immune function, emphasizing the need to investigate how hypoxia-driven metabolic shifts rewire T-cell metabolism and whether therapeutic strategies can reverse immune suppression and prevent neoplastic transformation. Thus, future research should focus on understanding the role of hypoxia-induced metabolic reprogramming on immune dysfunction, therapeutic resistance, and tumor cell survival. Investigating how metabolic pathways in GBM adapt to heterogeneous TME—across distinct tumor zones, nutrient availability, ECM, and responses to therapies—will provide critical insights into the metabolic crosstalk, helping identify potential therapeutic targets to disrupt tumor growth and immune evasion.

Amino acids and lipids are crucial nutrient sources for GBM and play significant roles in tumor progression and the remodeling of the TME, but the underlying mechanisms remain poorly understood. For example, the precise role of glutamine-derived α-ketoglutarate in tumor progression, the mechanism by which tryptophan metabolism-induced receptor activation modulates the immune response, and the connection between arginine deficiency, T cell dysfunction, and enhanced GBM progression all warrant further investigation. Lipid metabolism influences macrophage polarization towards a pro-tumorigenic phenotype and influences immune cell infiltration, especially in hypoxic niches where lipid-laden macrophages or foam cells may contribute to energy provision and immune suppression. Thus, understanding this relationship could lead to innovative therapies aimed at reprogramming macrophages to restore their anti-tumor functions and mitigate tumor progression. Along the same vein, metabolic support provided by TAAs to GBM cells, particularly through the transfer of cholesterol and mitochondria, is an emerging area of interest with promising therapeutic potential. Disrupting this metabolic crosstalk between TAAs and GBM cells, especially GSCs, could inhibit tumor growth and provide new treatment strategies. GSCs preferentially metabolize lipids for energy, yet the mechanisms driving this metabolic shift remain poorly understood. Thus, understanding the role of TAA-mediated cholesterol and mitochondrial transfer, as well as how these processes contribute to metabolic adaptation, including autophagy-mediated fatty acid release and mitochondrial energy production, could unlock new ways to disrupt the TME and enhance the efficacy of GBM therapies.

Recent studies have highlighted the significant relationship between GBM TME-induced metabolic rewiring and immune infiltration in driving GBM pathogenesis. However, critical knowledge gaps exist. Immune cell exhaustion is a prominent factor that allows tumor cells to evade immune surveillance and continue to propagate. While the role of co-inhibitory receptors such as PD-1 and TIM-3 to overcome immune cell exhaustion is well established, the precise molecular mechanisms driving these dysfunctional phenotypes are still poorly understood. To overcome immune cell exhaustion, a comprehensive multi-omics approach in both bulk and single-cell settings will be crucial to elucidate the metabolic profiles and shifts occurring within distinct immune cell subsets. This approach will unveil therapeutic reprogramming strategies to target immune cell metabolism and help restore immune function. By studying cross-cell metabolomics, researchers can better understand and mitigate the nutrient competition between the immune and GBM cells within the TME. This approach will allow researchers to engineer immune cells, enhancing their metabolic potential to survive and function under nutrient-limited conditions.

In summary, GBM is a multi-faceted and multi-component disease with interrelated crosstalk between the cells and TME. Thus, to effectively address this multimodal disease, combination therapies targeting multiple aspects of GBM’s pathogenesis will likely yield enhanced treatment efficacy. For example, combining agents that induce metabolic reprogramming with immune checkpoint inhibitors can provide a two-pronged approach to enhance anti-tumor immunity. This strategy would address the metabolic drivers of immune suppression and overcome the tumor’s immune evasion mechanisms. Several research studies have showcased the therapeutic potential of inhibiting LDH to revert immune suppression and tumor growth. As such, combination therapies may synergistically improve efficacy by neutralizing the GBM TME and restoring the effector functions of T cells and macrophages.

## 5. Conclusions

Metabolic rewiring plays a critical role in driving GBM progression and exacerbating immune evasion. A deeper understanding of the dynamic and convoluted interplay between tumor metabolism and immune cell function is essential for developing novel therapeutic strategies. Specifically, targeting key metabolic pathways—such as lactate production and lipid metabolism—alongside immune checkpoint inhibition offers a promising approach to enhancing anti-tumor immunity and overcoming treatment resistance. Further research is crucial to unravel the molecular mechanisms underlying these interactions and to optimize combination therapies that can restore immune surveillance, ultimately improving clinical outcomes for GBM patients.

## Figures and Tables

**Table 1:** Impact of metabolic rewiring on the immune cells in the GBM TME and current therapeutic interventions.

Immune Cell	Impact of Metabolic Rewiring	Therapeutic Intervention
T cells	○ OXPHOS to glycolysis, driven by mTORC signaling [140]. ○ Metabolic suppression of cytolytic activity due to tumor-induced TME remodeling [141].	○ Lipid-induced mitochondrial rewiring to maintain T cell adaptability [183]. ○ mTOR inhibition (e.g., rapamycin, Apitolisib) to restore T cell function [184,185]. ○ Engineered PGC-1α to enhance mitochondrial biogenesis [186]. ○ CD47 blockade to enhance cytotoxicity [187].
Tregs	○ Rely on glycolysis in low-glucose, high-lactate environments [142]. ○ Suppress anti-tumor immunity by sustaining immune-suppressive phenotype [142,143,144].	○ Disrupt glycolytic pathways and mTORC signaling using antisense oligonucleotides or mTORC inhibitor targeting [188,189]. ○ TLR8-mediated reprogramming to impair pro-tumorigenic function [190].
Microglia	○ Oxidative stress disrupts cholesterol metabolism, impacting antigen-presenting abilities, nurturing immunosuppressive function [164,165].	○ Genetic targeting of *NR4A2* enhances microglial plasticity and antigen presentation [163]. ○ Inhibition of SQLE (e.g., terbinafine) improves efficacy of immune checkpoint therapies [163].
TAMs	○ Lactate production leads to acidification of the TME, supporting TAM polarization [191].	○ Target MCT4 to inhibit lactate efflux [192]. ○ Use nanosheets, RNAi nanoparticles, and other catalysts to reduce lactate levels and promote immune activation [193,194,195,196].
LLM/TAF	○ LLM recycles myelin to provide lipids to GBM cells via LXR/ABCA-1 pathway [166]. ○ TAF engulfs EV and necrotic debris from GBM cells acquiring high levels of lipids [161]. ○ Lipid-loaded niche augments hypoxic response contributing to immune suppression [160,162].	○ Target lipid metabolism (lipid droplet formation, fatty acid oxidation, cholesterol metabolism [161,197,198]). ○ Inhibit HIF-1α stabilization with nanoplatforms [199].
TIDC	○ L-arginine-depravation affects the ability to activate effective anti-tumor immunity [173].	○ Supplement L-arginine to enhance dendritic cell function [200]. ○ Enhance activation and function through dendritic cell-based immunotherapies [201,202].
Astrocytes	○ Act as key players in the TME, transferring metabolic resources (e.g., cholesterol and mitochondria) to tumor cells [174,175,176,177]. ○ Metabolic rewiring to support tumor progression and immune evasion [175,178,183].	○ Inhibit cholesterol efflux from tumor-associated astrocytes to promote tumor regression [174]. ○ Target astrocytic transformation via EVs and tumor microtube-like structures for therapeutic intervention [192].

## Data Availability

No new data were created or analyzed in this study. Data sharing is not applicable to this article.
